# Recrystallization-Controlled Strength Evolution and Prediction of Compacted Rock-Salt Fills

**DOI:** 10.3390/ma19183975

**Published:** 2026-09-19

**Authors:** Liyang Wang, Ziliang Dong, Ruiling Feng, Feng Chen, Pengcheng Wang, Zhenyu Liu, Tengfei Wang

**Affiliations:** 1 Department of Geotechnical Engineering, State Key Laboratory of High-Speed Railway Track System, Beijing 100081, China; wangliyang@rails.cn (L.W.);; 2 Railway Engineering Research Institute, China Academy of Railway Sciences Corporation Limited, Beijing 100081, China; 3 School of Civil Engineering, Beijing Jiaotong University, Beijing 100044, China; 4 Key Laboratory of High-Speed Railway Engineering (Ministry of Education), School of Civil Engineering, Southwest Jiaotong University, Chengdu 610031, China

**Keywords:** rock salt, compacted fill, water content, unconfined compressive strength, recrystallization bonding, brine concentration, railway subgrade, salt-lake region

## Abstract

Locally available rock salt is a potential fill material for infrastructure construction in arid salt-lake regions, where conventional soil and aggregate resources are often scarce. However, its mechanical performance is strongly affected by water migration, brine concentration, salt dissolution, and possible recrystallization-induced bonding. This study investigates the unconfined compressive strength (UCS) of compacted rock-salt fills under different water contents, initial brine contents, relative compaction levels, and brine concentrations. The results show that UCS increases nonlinearly as water content decreases during air drying, with slow strength development at the early drying stage followed by rapid strength gain at low water contents. This behavior is consistent with a transition from capillary liquid bridges to stronger salt-related interparticle bonding during brine evaporation and possible recrystallization. The effect of initial brine content is state-dependent: higher initial brine content reduces UCS in the as-compacted state but markedly increases UCS after drying to constant mass, suggesting enhanced interparticle bonding after drying. Under constant-mass air-dried conditions, UCS first increases and then decreases with increasing relative compaction, reaching a peak of approximately 8.7 MPa at a relative compaction of 0.99. Lower brine concentration reduced the UCS of dried specimens, indicating that saturated brine may be more favorable for specimen preparation and field compaction under dry salt-lake conditions. An empirical UCS prediction equation incorporating water content, dry density, initial brine content, and brine concentration was developed and validated, giving good agreement with the test results with R^2^ = 0.91. The findings provide a material-level basis for evaluating and controlling compacted rock-salt fills under controlled dry conditions, while further durability and field validation are required before extending the results to long-term railway subgrade performance.

## 1. Introduction

In salt-lake regions of western China, conventional subgrade fill materials such as soil, gravel, and crushed rock are often scarce, and their long-distance transportation can substantially increase construction cost and environmental burden [1,2,3]. In contrast, rock salt is locally abundant in many dry salt-lake areas and has already been used in road and railway subgrade engineering [1,2,3,4]. Under dry and stable environmental conditions, compacted rock-salt fills exhibit relatively high strength because salt crystals provide interparticle bonding and contribute to the integrity of the compacted particle skeleton [3,5,6,7,8]. Therefore, the use of locally available rock salt as railway subgrade fill is of practical significance for infrastructure construction in arid salt-lake regions.

However, compacted rock-salt fills differ fundamentally from ordinary granular fills. Their engineering behavior is governed by dissolution, recrystallization, brine migration, damage evolution, and salt-crystal bonding [5,9,10,11,12,13,14]. Exposure to water or low-concentration brine dissolves salt particles, weakens interparticle contacts, and reduces the integrity of the compacted structure [10,11,12]. By contrast, during air drying, brine evaporation and salt precipitation drive recrystallization and re-cementation between particles, leading to strength recovery or strength enhancement [13,14,15,16,17]. Studies on cohesive granular media and salt crystallization in porous materials further show that drying-induced crystallization transforms liquid bridges into solid salt bridges, modifies the pore-scale structure, and controls the mechanical response of granular or porous systems [15,16,17]. For compacted rock-salt fills, macroscopic unconfined compressive strength (UCS) is therefore a direct engineering manifestation of these micro-scale bonding and debonding processes.

Because of this strong dependence on moisture-related processes, previous studies have quantified the engineering behavior of rock-salt fills under different compaction and water-exposure conditions. Existing work has mainly focused on compaction characteristics, the California bearing ratio, resilient modulus, immersion-induced degradation, water retention behavior, and water migration in rock-salt subgrades [1,2,3,4]. In particular, Cheng et al. [3] developed prediction models for the CBR and resilient modulus by considering brine content, the compaction degree, and immersion time. Their study provides an important basis for evaluating bearing performance and immersion-related degradation. Nevertheless, these indices mainly characterize load-bearing response under compaction and immersion conditions, and they do not directly describe UCS development during post-compaction air drying.

UCS is a practical index for evaluating the integrity and load-bearing capacity of cemented or weakly cemented fill materials, and it is widely used to develop empirical or data-driven strength prediction models for rock and rock-like materials [18,19,20,21,22]. For compacted rock-salt fills, UCS is particularly suitable for capturing the competition between water-induced weakening and recrystallization-induced bonding [13,14,15,16,17]. This competition depends on two related but distinct moisture variables: initial brine content, which controls lubrication, particle rearrangement, and the potential amount of crystallizable salt during compaction; and transient water content during drying, which controls water loss, brine concentration, salt precipitation, and the progressive formation of salt-crystal bonds. The air-drying stage after brine mixing and compaction is therefore a critical period during which the compacted particle skeleton evolves from a weak, moisture-sensitive structure into a cemented structure with higher integrity. For railway subgrades, this evolution is important because baseline UCS and structural integrity directly affect the ability of the fill layer to resist repeated cyclic and dynamic loading during long-term railway operation. If this stage is neglected, the strength reserve and deformation resistance of rock-salt subgrades may be misjudged, leading to uncertainty in compaction control, drainage protection, and track-support performance. However, a quantitative framework linking UCS to the compaction state, brine condition, and drying-induced water loss remains insufficiently established.

In this study, laboratory UCS tests were conducted on compacted rock-salt fills under varying compaction states, brine conditions, and environmental drying stages. The objectives were to identify the main factors controlling UCS, clarify the strength evolution mechanism during air drying, establish an empirical prediction equation incorporating key material and preparation parameters, and provide a quantitative basis for the design and construction control of rock-salt fills in railway subgrade engineering.

## 2. Materials and Test Methods

This section describes the rock-salt material, its physical and mineral characteristics, and the UCS testing program designed to isolate the effects of water content, initial brine content, the compaction degree, and brine concentration.

### 2.1. Test Materials

**Figure 1:**
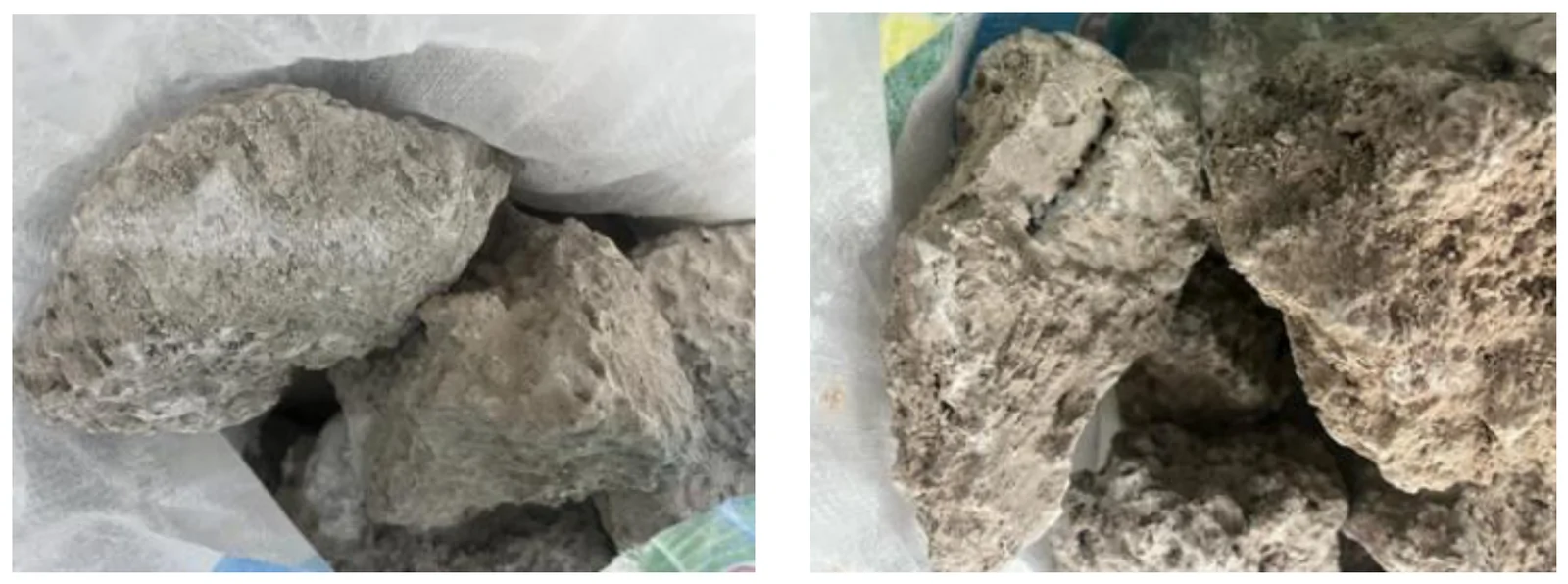
Representative rock salt blocks collected from the field.

The rock salt used in this study was collected from a salt-lake region in Northwest China, as shown in Figure 1. During sampling, well-consolidated rock salt within 0–50 cm below the ground surface was collected. Visual inspection showed that the rock salt blocks were relatively dense, gray both internally and externally, and relatively hard, with distinct fissures observed locally.

The basic physical properties of the rock salt were determined following the corresponding standards. The natural air-dried water content was measured in accordance with ASTM D2216/D2216M [23], the particle density was determined using a gas pycnometer in accordance with ASTM D5550 [24], and the water-insoluble matter content was measured in accordance with ISO 2479:1972 [25]. The results indicate that the natural air-dried water content, particle density, and water-insoluble matter content of the rock-salt fill material were 0.82%, 2.32 g/cm^3^, and 24.69%, respectively.

The mineral compositions of the rock salt and evaporated brine crystals were determined by X-ray powder diffraction in accordance with EN 13925-2:2003 [26]. The results are listed in Table 1. The mineral percentages listed in Table 1 were obtained from semi-quantitative XRD analysis based on the relative intensities of characteristic diffraction peaks(XRD measurements were performed on a Bruker D8 Advance X-ray diffractometer (Bruker AXS SE, Karlsruhe, Germany)). For the saturated brine, the mineral composition was analyzed using crystals obtained by evaporating the saturated brine.

The results show that the rock salt used in this study was mainly composed of halite, namely sodium chloride. The saturated brine used in the tests was prepared by fully dissolving rock salt fines with a particle size ≤ 1 mm in pure water. The measured total dissolved solids was 308.92 g/L, and the brine concentration was 24.95 wt%. The saturated brine was mainly composed of halite and K-bearing salts.

The measured water-insoluble matter content of the rock-salt fill was 24.69%, which is generally consistent with the presence of non-halite minerals identified by XRD. This insoluble fraction may influence strength development in two ways. On the one hand, minerals such as quartz, calcite, plagioclase, chlorite, gypsum, and illite may act as relatively stable skeleton or filler components, contributing to interparticle friction, contact roughness, and structural stability after compaction. On the other hand, these minerals do not directly participate in NaCl dissolution–precipitation bonding. A relatively high insoluble fraction may reduce the amount of recrystallizable halite and locally interrupt the continuity of salt-crystal bridges. Therefore, the drying-induced strength development should be interpreted as the combined result of mechanical interlocking/friction from the granular skeleton and possible recrystallization bonding of soluble salts. Because the insoluble fraction was not varied in this study, its quantitative influence on UCS requires further investigation.

### 2.2. Experimental Program

Previous studies indicate that water content, initial added brine content, dry density, and brine concentration are the key factors controlling the physical and mechanical properties of rock-salt fill materials. To evaluate the effects of these factors, UCS tests were conducted on rock-salt fill materials under different conditions. The experimental program is shown in Table 2.

**Figure 2:**
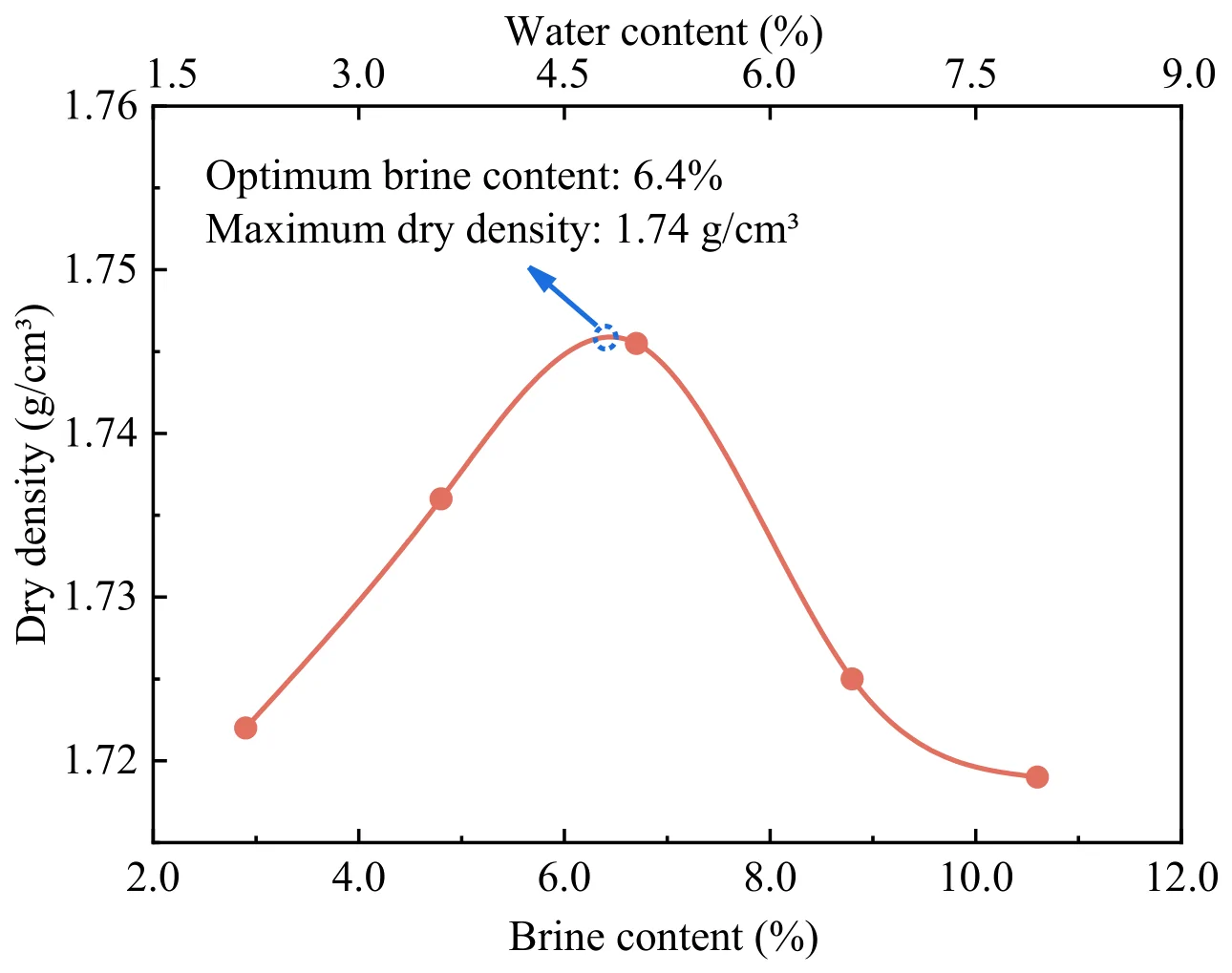
Compaction curve of rock salt.

The modified compaction test was conducted in accordance with ASTM D1557/D1557M [27], and the compaction curve is shown in Figure 2. The optimum brine content and maximum dry density were determined to be 6.4% and 1.74 g/cm^3^, respectively. Accordingly, degrees of compaction of 0.90, 0.93, 0.95, 0.99, and 1.06 were adopted in the experimental program, corresponding to dry densities of 1.57, 1.62, 1.66, 1.73, and 1.85 g/cm^3^, respectively. It should be noted that the compaction degrees of 0.99 and 1.06 were determined from a porosity-based specimen design. Based on the measured particle density of 2.32 g/cm^3^ and the maximum dry density of 1.74 g/cm^3^, K=0.93 corresponds to a porosity of approximately 30% according to $n=1-\rho_{d}/(G_{s}\rho_{w})$. Therefore, two lower target porosities of 25% and 20% were further designed, corresponding to compaction degrees of 0.99 and 1.06, respectively.

The tests were conducted using a WDW-50E computer-controlled universal testing machine (Jinan Hensgrand Instrument Co., Ltd., Jinan, China), a hydraulic specimen preparation and demolding machine, molds, an electronic balance, and other auxiliary apparatus.

**Figure 3:**
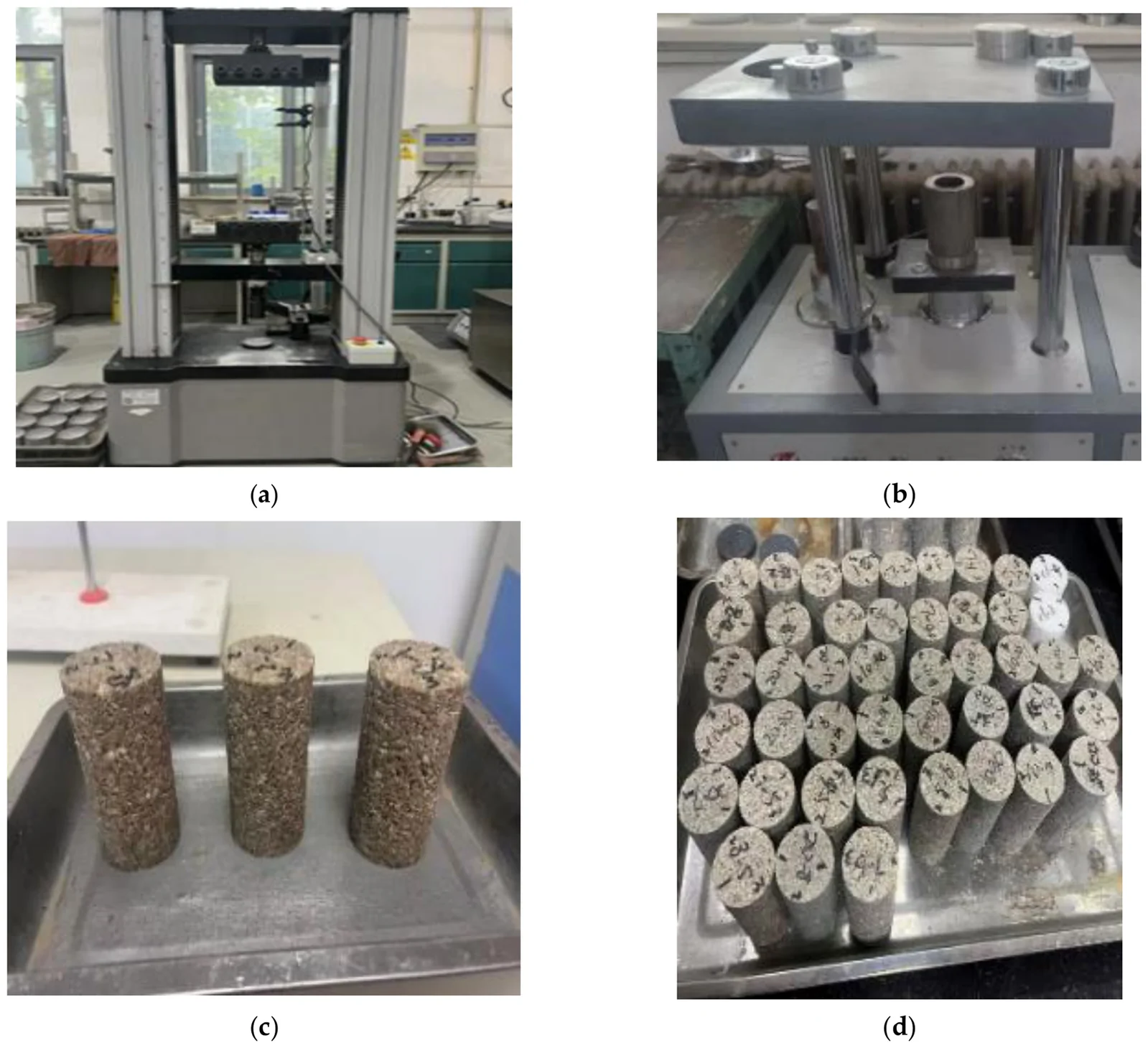
Test equipment and rock salt specimens: (**a**) universal testing machine; (**b**) hydraulic compaction and demolding apparatus; (**c**) specimens during air drying; and (**d**) specimens after reaching constant mass.

The test procedures were as follows: (1) Natural rock salt was crushed, ground, and sieved, with the maximum particle size controlled below 5 mm. (2) Brine with the specified brine concentration and brine content was added, and cylindrical specimens with a diameter of 50 mm and a height of 100 mm were prepared at the target degree of compaction. (3) The specimens were placed indoors for air drying, and different air-drying times were adopted to obtain specimens with different water content levels. The indoor air-drying conditions were measured as 25–28 °C in temperature and 20–30% in relative humidity. (4) After air drying for the specified time, as listed in Table 2, the specimens were subjected to UCS tests at a loading rate of 1 mm/min and a sampling frequency of 1 Hz. The test equipment and rock salt specimens are shown in Figure 3.

## 3. Results and Discussion

This section presents the experimental results and interprets the strength evolution of compacted rock salt specimens during drying, with emphasis on water loss, recrystallization bonding, initial brine content, brine concentration, and the compaction degree.

### 3.1. Mass and Water-Content Changes During Air Drying

**Figure 4:**
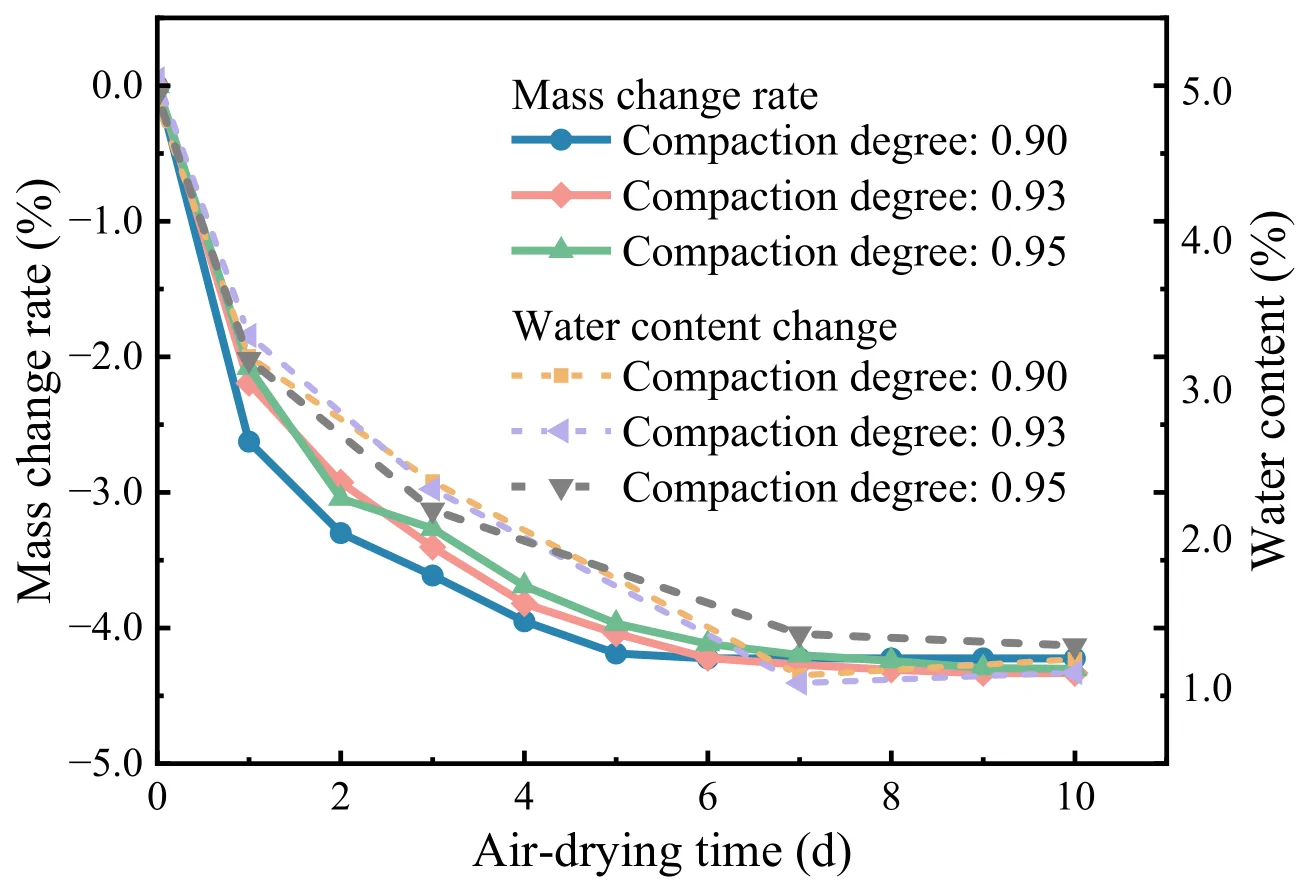
Changes in mass and water content during air drying.

The mass variation in the specimens in Group 1 with air-drying time is shown in Figure 4. The mass change rate was defined as the relative change in specimen mass after *t* days of air drying with respect to the initial mass. It was calculated by Equation (1).

(1) $$R_{m}=\frac{m_{t}-m_{0}}{m_{0}}\times 100\%,$$

where *R_m_* is the mass change rate, *m*_0_ is the initial mass of the specimen, and *m_t_* is the mass of the specimen after air drying for *t* days.

As shown in Figure 4, specimens with different degrees of compaction exhibit similar mass variation trends with air-drying time, although the magnitudes of mass loss differ. In the early stage of air drying, approximately 0–2 days, the specimen mass decreases rapidly. As air-drying time increases, the mass loss rate gradually decreases and then tends to stabilize. This behavior is mainly attributed to the relatively high water content of the specimens after preparation, which leads to rapid water evaporation in the early stage. With continued air drying, most residual water remains inside the specimens and evaporates more slowly, thereby reducing the mass loss rate. The water content shows a similar variation trend, indicating that the two trends are mutually consistent.

In the early stage of air drying, the specimen with a degree of compaction of 0.90 exhibits the highest mass loss rate. This indicates that a lower degree of compaction produces a looser structure, allowing water to migrate outward and evaporate more easily. In contrast, the specimens with degrees of compaction of 0.93 and 0.95 have smaller pores and lower pore connectivity, resulting in a lower water migration and evaporation rate, a slower mass change in the early stage of air drying, and a slightly longer time to reach a stable mass. Constant mass was considered to have been reached when the mass change was ≤0.1%/24 h [28].

Under these test conditions, after 10 days of air drying, the mass change rate and water content of all specimens with different degrees of compaction stabilized at approximately −4.3% and 1.1~1.3%, respectively. This indicates that the specimens had nearly reached a constant-mass air-dried state.

As discussed above, the variations in mass and water content are mutually consistent. Therefore, the easily measurable mass change in the specimens was used as the criterion in this study. When the specimen mass reached a constant value, the specimen was considered to have reached a constant-mass air-dried state.

However, mass change is less intuitive as a unified indicator for subsequent analysis, and using it directly is not sufficiently concise. Because mass change and water content exhibit consistent trends during air drying, water content was adopted as the unified indicator for the subsequent analysis, providing a more general and consistent basis for comparison.

As water content is a key parameter in the subsequent analysis, its accuracy was further verified by calculating the theoretical water content and comparing it with the measured value. The theoretical water content was calculated by Equation (2), and the calculated and measured results are listed in Table 3.

(2) $$w_{t}=\frac{m_{w}}{m_{s}}+w=\frac{\left(m_{t}-m_{s0}\right)\left(1-C\right)}{m_{s0}+\left(m_{0}-m_{t}\right)C}\times 100\%+w,$$

where *w_t_* is the water content at air-drying time *t*; *m_w_* is the mass of water in the specimen at air-drying time *t*; *w* is the natural air-dried water content of the specimen; *m_s_* is the specimen dry mass at air-drying time *t*; mt is the specimen mass at air-drying time *t*; *m_s_*_0_ is the initial dry mass of the specimen; *m*_0_ is the initial total mass of the specimen; and *C* is the brine concentration.

In Table 3, the theoretical water content was calculated as the sum of the inherent water in the natural air-dried rock salt and the water introduced by the brine during specimen preparation. The former was calculated from the natural air-dried water content of the specimen, whereas the latter was calculated based on the brine concentration. It should be emphasized that this calculation is not an independent validation of the measured water content, because it is based on several simplifying assumptions, including uniform brine distribution, unchanged brine concentration during specimen preparation, negligible material loss during drying, and the assumption that the measured mass loss is mainly associated with water evaporation. Therefore, the comparison in Table 3 is used only as a consistency check between the water-content measurements and the mass-balance calculation.

As shown in Table 3, the theoretical and measured water contents at the corresponding air-drying times are in good agreement, with only small differences between them. The difference relative to the theoretical value ranged from −0.21% to 0.50%, and all differences were within 0.5%, which falls within the allowable error range for water content testing specified in the standard. Therefore, this consistency supports the use of measured water content as the main state variable for subsequent UCS analysis. The discrepancy in water content may be attributed to two main factors. First, local heterogeneity in rock salt may cause the natural water content to vary among different portions, resulting in experimental error. Second, during brine preparation, variations in temperature and in the types of dissolved salts may lead to differences in brine concentration, thereby causing deviations between the theoretical and actual water contents.

Based on the variations in mass and water content during air drying, the mass and water content of the rock salt specimens under the present test conditions tended to stabilize after 10 days of air drying.

### 3.2. UCS Evolution During Air Drying

Air drying involves continuous internal water migration and evaporation within the specimen, which is macroscopically reflected by changes in water content. In addition, the degree of compaction affects the internal compactness of the specimen and the evolution of water content during air drying. Therefore, this section focuses on the effects of water content and the degree of compaction on the UCS of rock salt specimens.

**Figure 5:**
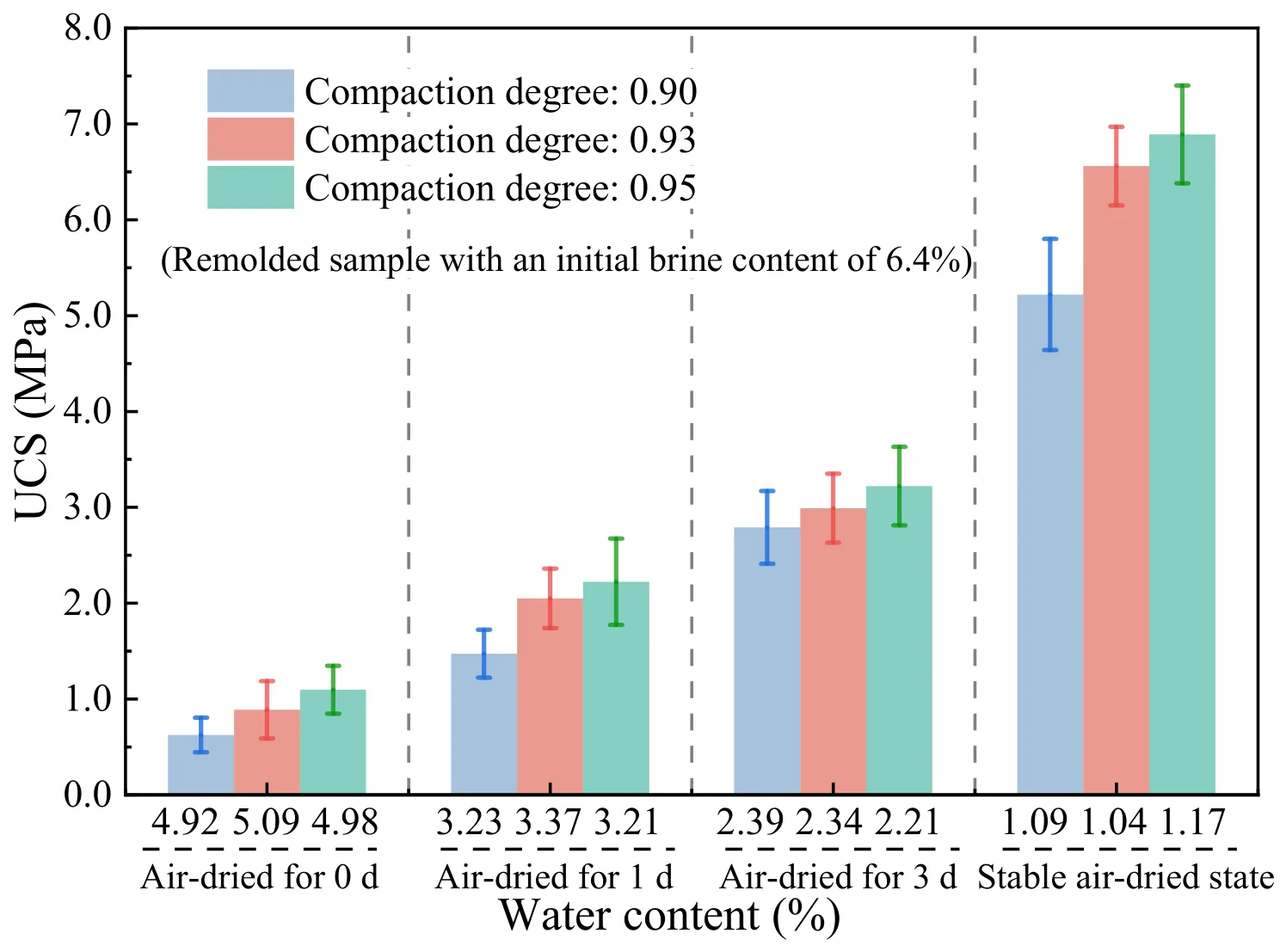
UCS of rock salt specimens with different degrees of compaction at various water contents.

The variation in UCS with water content under different degrees of compaction is shown in Figure 5. Water content has a significant effect on the UCS of rock salt specimens, and similar trends are observed under different degrees of compaction. As air drying proceeds, the water content of the specimens gradually decreases, whereas the UCS increases markedly. For example, at a degree of compaction of 0.93, the UCS is 0.89 MPa at 0 d of air drying and increases to approximately 6.56 MPa after drying to constant mass. In addition, at the same air-drying time and similar water content, higher degrees of compaction generally correspond to higher UCS.

**Figure 6:**
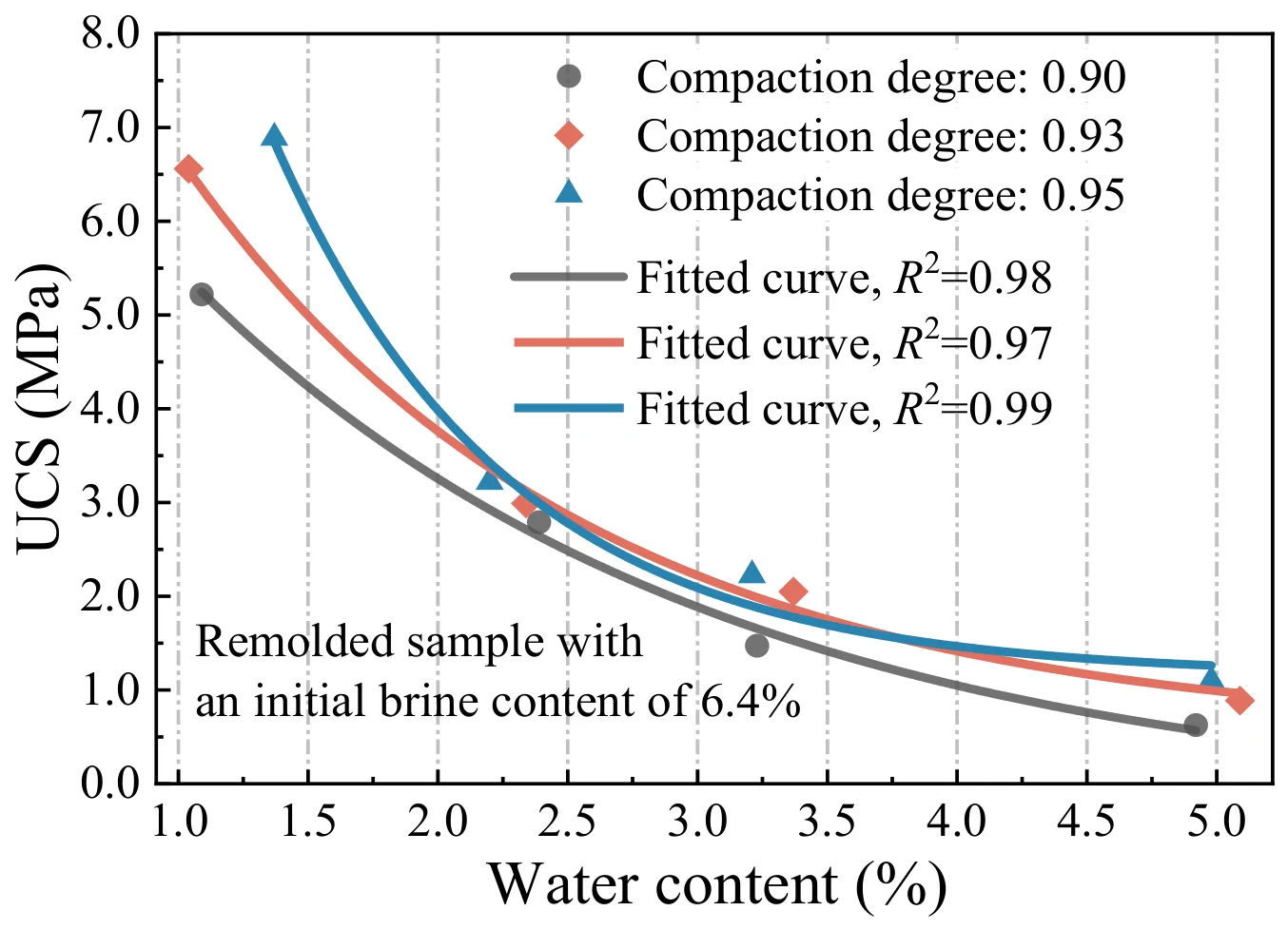
Relationship between UCS and water content for rock salt specimens with different degrees of compaction.

The experimental relationships between UCS and water content for specimens with different degrees of compaction were plotted and fitted, as shown in Figure 6, and the corresponding fitting equation and parameter are given in Equation (3) and Table 4. 

It should be noted that the fitting form adopted in this study is an exponential empirical equation commonly used to describe the water sensitivity of soft rocks. This exponential relationship reflects the nonlinear increase in UCS during the drying process, particularly the rapid strength enhancement observed at low moisture contents. For other empirical functions, such as linear functions, a linear function mainly represents the overall decreasing trend in UCS with increasing moisture content, but it cannot adequately capture the accelerated strength gain observed within the low-moisture-content range. Therefore, the exponential form was selected as the fitting equation.

The results show that all *R*^2^ values are no less than 0.97, indicating that Equation (3) provides a good description of the relationship between UCS and water content of rock salt specimens upon air drying.

(3) $$\begin{array}{l} q_{0.90}=9.43e^{-0.49\omega }-0.27\\ q_{0.93}=11.77e^{-0.64\omega }+0.52\\ q_{0.95}=26.03e^{-1.11\omega }+1.16 \end{array},$$

where *q*_0.90_, *q*_0.93_, and *q*_0.95_ denote the UCS (MPa) at degrees of compaction of 0.90, 0.93, and 0.95, respectively, and *ω* denotes the water content (%).

As shown in Figure 6 and Equation (3), the UCS of rock salt specimens increases nonlinearly as water content decreases during air drying. Overall, the relationship follows an exponential trend, with a relatively slow increase in the early stage and an accelerated increase in the later stage. For example, for the specimen with a degree of compaction of 0.93, the strength increases by approximately 1.3 MPa when the water content decreases from 5.1% to about 3.4%. When the water content further decreases from about 2.3% to 1.0%, the strength increases by approximately 3.57 MPa. This indicates that the UCS of rock salt is more sensitive to changes in water content within the low-water-content range.

This behavior is generally consistent with the typical strength–water content relationship observed in soft rocks such as mudstone and sandstone [18]. However, most existing studies on soft rocks have focused on intact rock specimens, whereas the specimens examined in this study were remolded rock salt specimens prepared by crushing, remixing, and compaction. Their internal structure is essentially a particle-contact skeleton structure. During air drying, the remolded rock salt still exhibits a strength increase pattern similar to that of soft rock, suggesting that drying-induced salt precipitation and possible recrystallization-induced re-cementation may occur during water loss.

From a mechanistic perspective, crushed and remolded rock salt can be regarded as an unsaturated granular material, and its self-healing process during air drying is essentially similar to the bonding process of unsaturated granular materials reported by Delenne [15]. In a saturated NaCl solution–sand particle system, Delenne [15] showed that interparticle connections in unsaturated granular materials transform from liquid bridge-dominated bonding to solid salt-bridge bonding during drying. The strength development can be divided into three stages. In the early stage, capillary bonding dominates, liquid bridges act between particles, and strength increases slowly. In the middle stage, crystallization bonding develops; interconnected crystalline bonds first form near the surface and then gradually extend inward, rapidly enhancing interparticle cohesion and accelerating strength growth. In the later stage, cementation and solidification dominate; the liquid phase is largely transformed into solid crystalline bonds, the overall specimen structure becomes stable, and the strength further increases. This process is consistent with the strength evolution of rock salt specimens observed in this study, namely a slow initial increase followed by rapid strength growth during air drying. Delenne [15] also noted that the liquid phase is difficult to completely transform into crystal bridges because residual water remains inside the sample, which is consistent with the test results presented in Section 3.5.

Studies on salt crystallization in porous materials further show that evaporation-driven crystallization can significantly modify the pore-scale structure and mechanical response, and that the resulting effect is closely related to crystallization kinetics and the interfacial properties among the salt solution, crystals, and porous matrix [16].

In addition, Han Jianwen et al. [29] reported that, after rock salt is crushed, mixed with saturated brine, and compacted, the saturated brine filling interparticle pores and fissures gradually precipitates and crystallizes during air drying. Crystallization may promote bonding among compacted rock-salt particles. Based on the UCS evolution and previous studies, the strength increase in rock-salt fill materials during air drying can therefore be interpreted as a possible structural reconstruction process associated with the gradual transformation from liquid capillary bridges to solid crystalline bonding. In the early stage of air drying, interparticle interaction may still be mainly influenced by liquid bridges, lubrication, and friction, and the strength increase is relatively limited. As water further evaporates, the pore solution gradually becomes supersaturated, leading to salt precipitation and crystallization. Solid salt bridges may gradually form and develop between particles, resulting in a marked increase in strength. When the specimens approach the constant-mass air-dried state, the internal cemented structure becomes progressively developed, and the specimens exhibit relatively high macroscopic UCS.

This behavior is markedly different from the structural deterioration commonly observed in soft rocks after water loss and air drying. For example, during the drying of red-bed soft rocks, clay minerals such as montmorillonite and illite undergo volumetric shrinkage upon dehydration. As a result, fissures and cracks that were previously filled by water-induced swelling reopen, porosity increases, structural integrity decreases, and strength tends to deteriorate [30]. In contrast, possible crystallization bonding may play an important role in the air-drying process of rock-salt fill materials, leading to pronounced strength recovery and enhancement and reflecting the favorable self-healing properties of rock salt.

In summary, the UCS of rock-salt fill materials increases significantly as water content decreases during air drying, exhibiting a nonlinear growth pattern characterized by a slow initial increase followed by accelerated growth. This behavior is interpreted as being related to drying-induced salt precipitation and possible recrystallization bonding during air drying, which promotes the gradual transformation of remolded rock salt from a loose granular assembly into a cemented structure with integrated bearing capacity. This self-healing effect enables rock-salt fill materials to macroscopically exhibit a strength evolution pattern similar to that of soft rock.

### 3.3. Effect of Initial Brine Content on UCS

**Figure 7:**
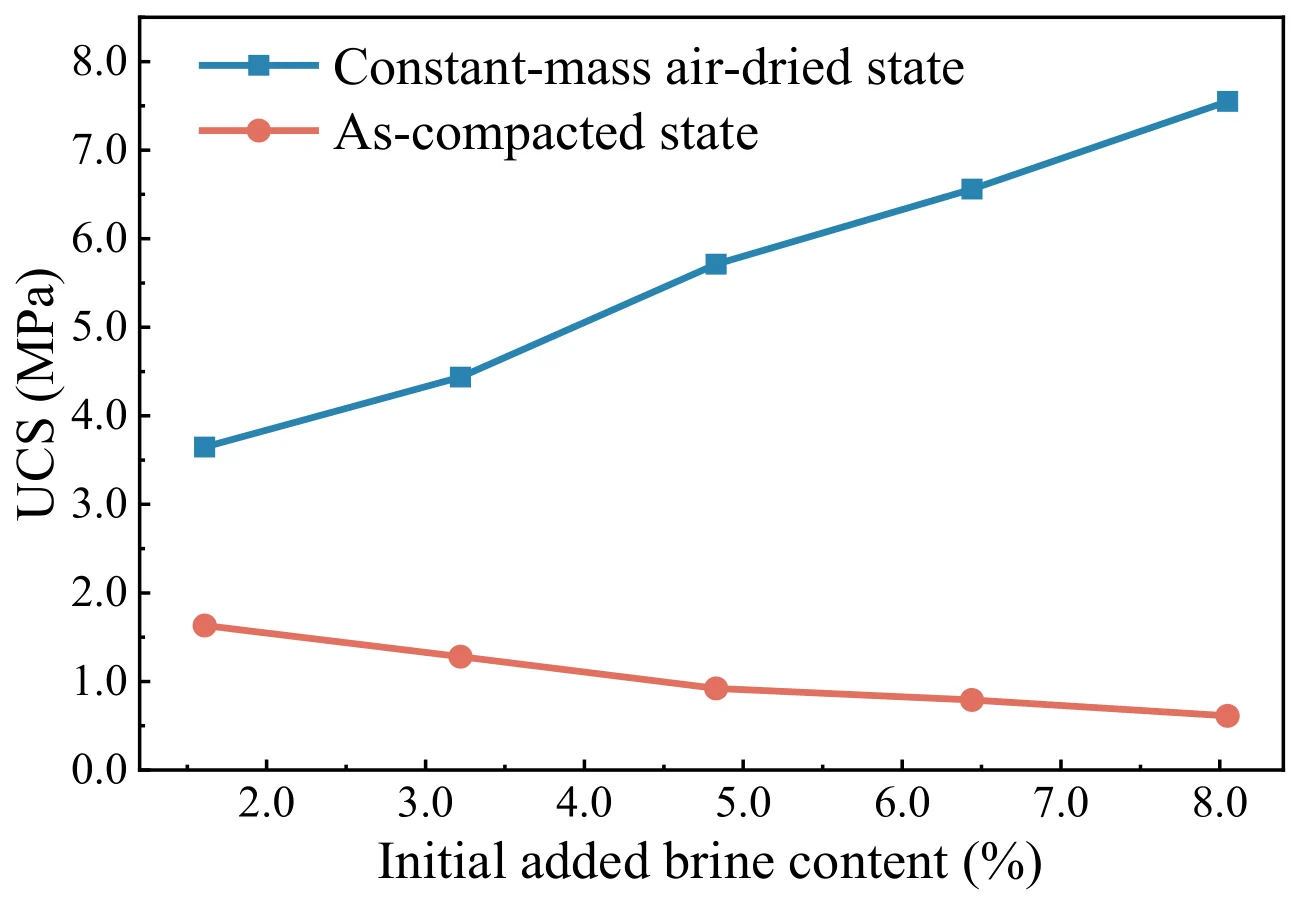
Effect of initial added brine content on the UCS of rock salt specimens in the as-compacted and constant-mass air-dried states.

The UCS test results of rock salt specimens with different initial added brine contents in the as-compacted and constant-mass air-dried states are shown in Figure 7.

As shown in Figure 7, in the as-compacted state, the strength of rock salt specimens decreases markedly as the initial added brine content increases. Compared with the specimen with an initial added brine content of 1.6%, the compressive strengths of specimens with initial added brine contents of 8.1%, 6.4%, 4.8%, and 3.2% decrease by approximately 63%, 51%, 42%, and 21%, respectively. This is mainly because the early-stage strength of rock salt in the wet state is primarily provided by interparticle interlocking and friction. A higher initial added brine content enhances the lubricating effect between particles, thereby weakening interparticle interactions and reducing the macroscopic strength of rock salt specimens.

In the constant-mass air-dried state (constant mass), the strength of rock salt specimens increases significantly with increasing initial added brine content. Compared with the specimen with an initial added brine content of 1.6%, the compressive strengths of specimens with initial added brine contents of 3.2%, 4.8%, 6.4%, and 8.1% increase by approximately 29%, 93%, 147%, and 223%, respectively. This is because the strength of rock salt after drying is mainly derived from salt recrystallization bonding. A higher initial added brine content promotes the precipitation of more salt crystals between particles after air drying, resulting in more extensive crystalline bonds and, consequently, a significant increase in macroscopic strength.

Therefore, the effect of initial added brine content on the UCS of rock salt specimens is strongly state-dependent. In the as-compacted state, UCS decreases with increasing initial added brine content, whereas in the constant-mass air-dried state, it increases significantly with increasing initial added brine content.

### 3.4. Effect of Brine Concentration on UCS

**Figure 8:**
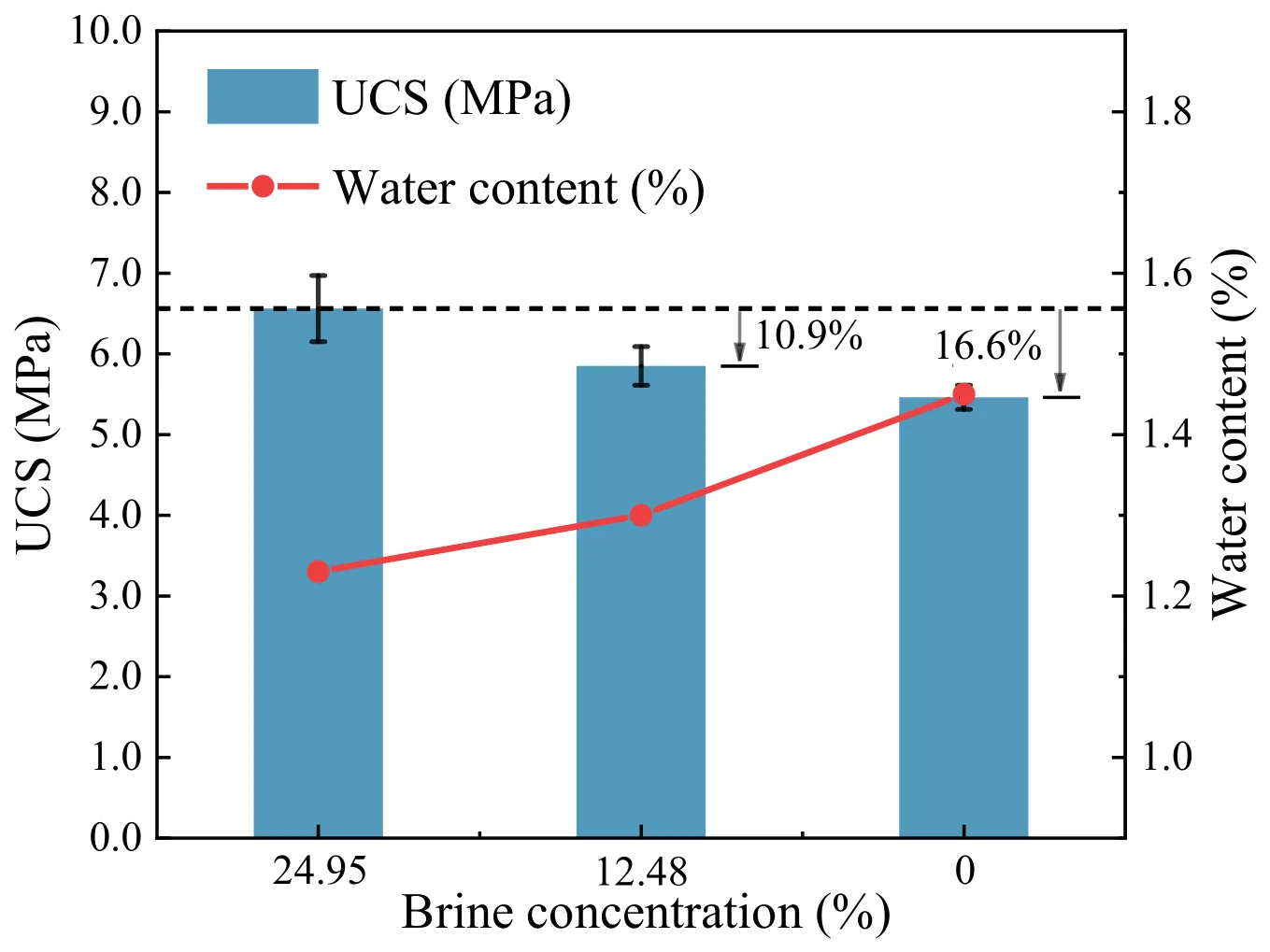
Effect of brine concentration on the UCS of rock salt specimens.

The UCS test results of rock salt specimens prepared with different initial brine concentrations are shown in Figure 8. During specimen preparation, the oven-dry density of specimens with different initial brine concentrations was kept constant to eliminate the influence of dry density differences after air drying on strength comparison.

As shown in Figure 8, in the constant-mass air-dried state, the strength of rock salt specimens decreases as the initial brine concentration decreases. Compared with saturated brine at 24.95 wt%, the UCS values of specimens prepared with half-saturated brine at 12.48 wt% and pure water decreased by approximately 10.9% and 16.6%, respectively, indicating a relatively small reduction. This is because, when unsaturated brine, especially pure water, is used for mixing and specimen preparation, salts in the rock salt dissolve into the water, forming saturated brine. On the one hand, this dissolution effect erodes the surface of rock salt particles and generates additional pores and defects. On the other hand, it may directly dissolve salt crystals originally present at interparticle contacts, separating particles or enlarging existing pores. This weakens effective interparticle contact and the nucleation conditions for subsequent crystalline bonding, ultimately leading to a slight decrease in the macroscopic strength of rock salt specimens.

### 3.5. Effect of Compaction Degree on UCS

**Figure 9:**
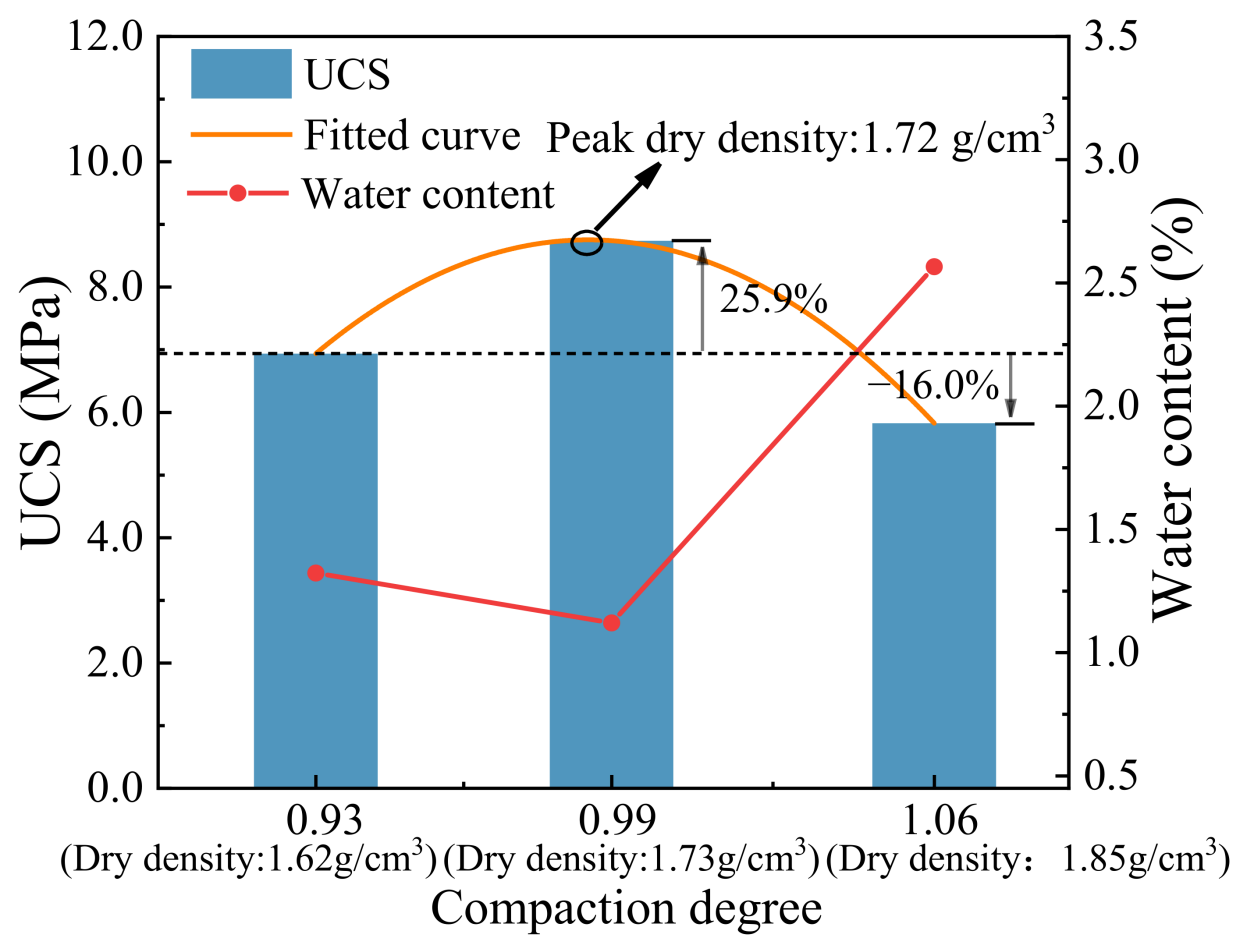
Effect of degree of compaction on the UCS of rock salt specimens in the constant-mass air-dried state.

The test results for degrees of compaction of 0.90, 0.93, and 0.95 were presented in Section 3.2; however, the differences among these degrees of compaction were relatively small. To further clarify the effect of the degree of compaction on the UCS of rock salt specimens, additional comparative tests were conducted with 0.93 as the reference degree of compaction. Degrees of compaction of 0.93, 0.99, and 1.06 were adopted, corresponding to dry densities of 1.62, 1.73, and 1.85 g/cm^3^, respectively. The test results are shown in Figure 9. The measured moisture contents of the specimens after reaching the air-dried constant-mass state are presented on the right-hand axis of Figure 9, together with the corresponding curve.

As shown in Figure 9, in the constant-mass air-dried state, the compressive strengths of rock salt specimens with different degrees of compaction are 6.9 MPa, 8.7 MPa, and 5.8 MPa, respectively. This trend differs from that of conventional fills: the strength does not increase monotonically with the degree of compaction, but instead first increases and then decreases. The peak UCS is approximately 8.7 MPa, corresponding to a degree of compaction of approximately 0.99 and a dry density of approximately 1.72 g/cm^3^.

**Figure 10:**
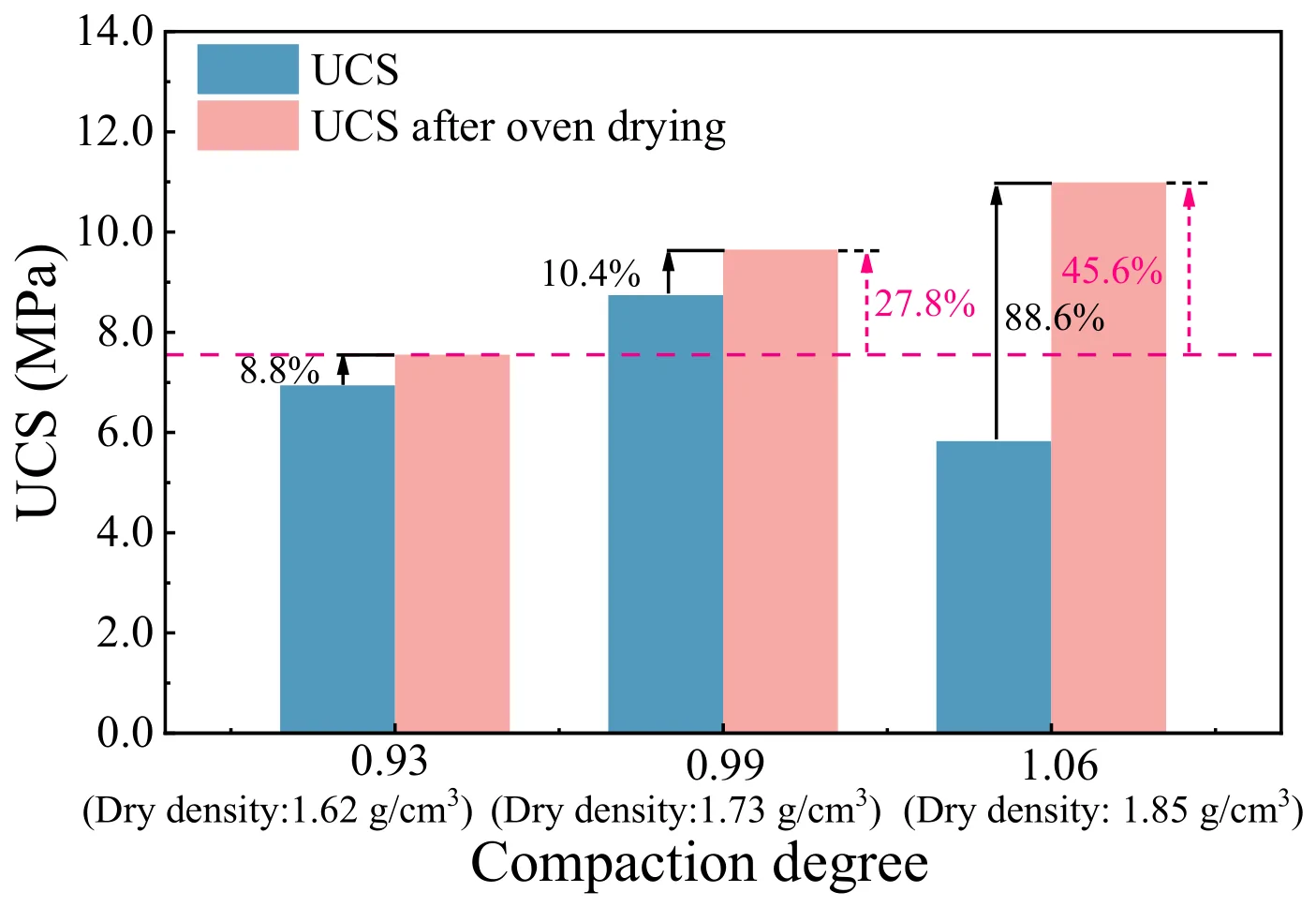
UCS of rock salt specimens before and after oven drying at different degrees of compaction.

This behavior is mainly because reaching constant mass does not necessarily indicate the same residual water content in rock salt specimens. In particular, under a high degree of compaction, the pores are finer and pore connectivity is lower, making residual brine more difficult to migrate outward. Consequently, specimens with different degrees of compaction may still have different residual water contents and incomplete crystallization processes even after reaching constant mass. Therefore, to exclude the effect of residual water differences, the specimens were further subjected to oven drying after reaching constant mass, and the test results are shown in Figure 10.

As shown in Figure 10, the UCS of all specimens increases after oven drying. The increase is most pronounced for the specimen with a degree of compaction of 1.06, reaching 88.6%.

This indicates that specimens with higher degrees of compaction are more likely to retain crystallizable residual brine during the air-drying stage. Oven drying promotes the complete evaporation of residual brine and induces crystallization at critical interparticle contact points, thereby strengthening salt-bridge bonding. In contrast, specimens with lower degrees of compaction contain less residual brine; therefore, the additional cementation induced by oven drying is limited, resulting in a smaller increase in strength. After the influence of residual water differences was further excluded, the strength of rock salt specimens showed a clear positive correlation with the degree of compaction.

Overall, in the constant-mass air-dried state, the UCS of rock salt specimens exhibits an apparent trend of first increasing and then decreasing with an increasing degree of compaction. However, after the influence of residual water differences was further excluded, the strength generally increased with the degree of compaction. This indicates that the effect of the degree of compaction on the UCS of rock salt specimens is strongly influenced by residual water content.

## 4. Prediction of the UCS of Rock-Salt Fill Materials

This section integrates the identified controlling factors into a UCS prediction framework, evaluates their relative importance, validates the empirical equation, and discusses engineering implications for using rock-salt fill materials in railway subgrades.

### 4.1. Relative Importance of Factors Affecting UCS

**Figure 11:**
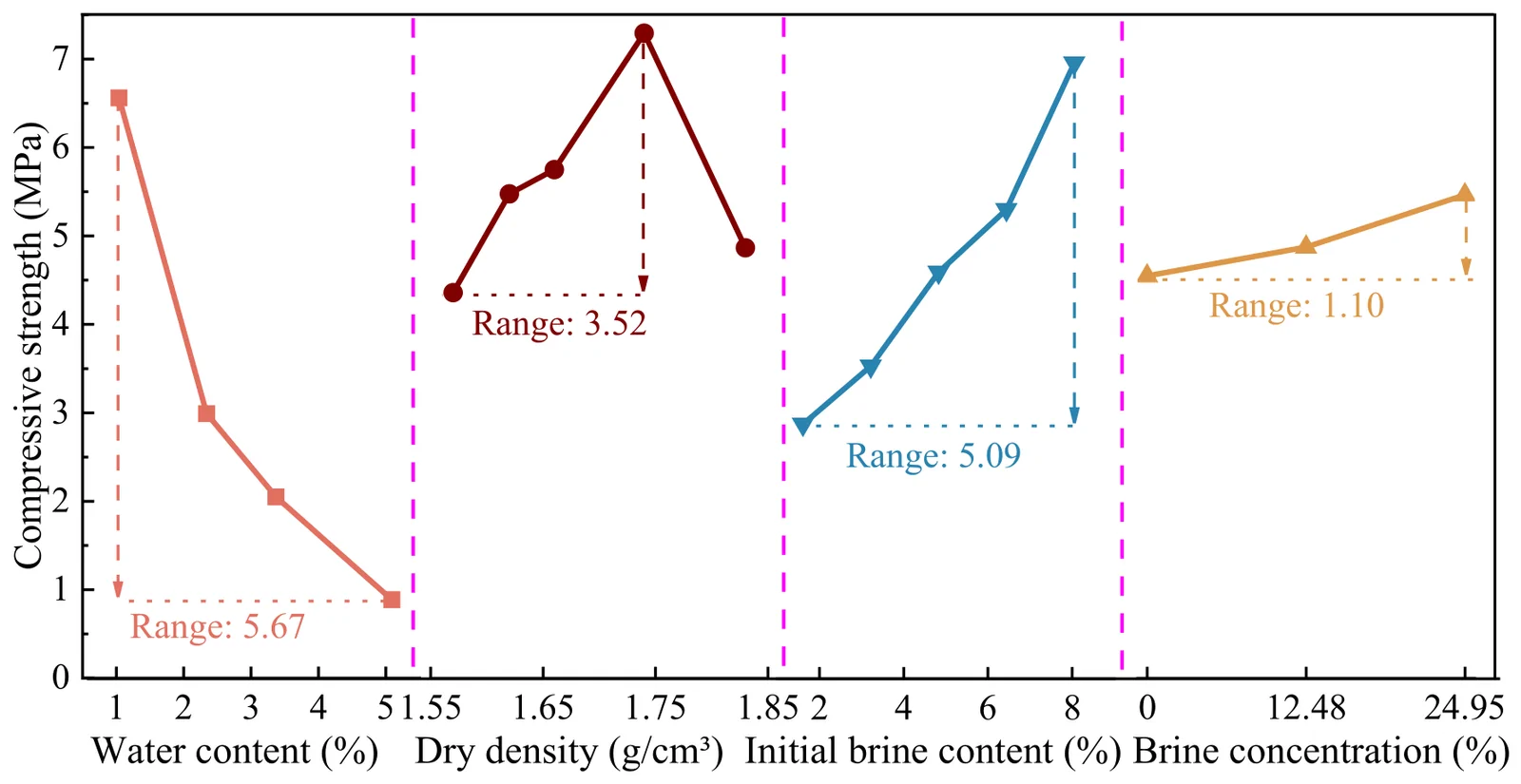
Range analysis of factors affecting the UCS of rock salt specimens.

To quantify the effects of water content, dry density (degree of compaction), initial added brine content, and brine concentration on the UCS of rock salt specimens, range analysis was performed using the experimental results described above. The results are shown in Figure 11.

As shown in Figure 11, the range values for water content, dry density, initial added brine content, and brine concentration are 5.67, 3.52, 5.09, and 1.10, respectively. Within the factor levels considered in this study, the relative influence of these factors on UCS follows the order: water content > initial added brine content > dry density (degree of compaction) > brine concentration. This indicates that water content has the most significant effect on the UCS of rock salt specimens, whereas the concentration of the brine used for specimen preparation has the weakest effect among the examined factors.

### 4.2. Empirical Prediction Equation for the UCS of Rock-Salt Fill Materials

Studies on the strength of rock salt remain limited. However, the strength evolution of rock salt during air drying shows a variation pattern similar to that of intact soft rock. Therefore, the empirical exponential equation proposed by Hawkins et al. and commonly used in soft rock studies was first adopted as the basic functional form [18]:

(4) $$q=ae^{-bw}+c$$

where *q* is the UCS (MPa), *w* is the water content (%), and *a*, *b*, and *c* are empirical parameters.

However, Equation (4) only accounts for water content and does not incorporate specimen conditions such as density. In addition, rock salt specimens are affected by specific factors such as brine concentration and initial added brine content, which are not represented in Equation (4). Therefore, the empirical equation needs to be modified.

Vásárhelyi [19] reported that the water sensitivity of sandstone strength depends largely on rock porosity and that strength follows a power–exponential relationship with porosity. Based on sandy mudstone, Guo Rui [31] further indicated that strength varies with density according to a power-law relationship. Accordingly, a density-related correction term associated with porosity was introduced into the empirical equation in this study.

The modified equation is expressed as follows:

(5) $$q={\left(\frac{\rho }{\rho_{s}}\right)}^{m}\left(ae^{-bw}+c\right)$$

where *q* is the UCS (MPa), *ρ* is the specimen density (g/cm^3^), *ρ_s_* is the particle density (g/cm^3^), *w* is the specimen water content (%), and *a*, *b*, *c*, and *m* are empirical parameters.

Equation (5) still does not account for the effects of rock-salt-specific variables, such as brine concentration and initial added brine content, on the UCS of rock salt. Therefore, the crystallized salt mass *S* was introduced to incorporate variations in specimen conditions. *S* represents the mass of salt crystals precipitated from pore brine during air drying of the specimen and is expressed as follows:

(6) $$\begin{array}{l} S=\rho VCw_{0}-\frac{\rho VC\left(w_{t}-w\right)}{1-C}=\rho VC\left(w_{0}-\frac{w_{t}-w}{1-C}\right)\\ =\rho VC\frac{w_{0}-w_{0}C-w_{t}+w}{1-C}=\frac{\rho VC}{1-C}\left(w_{0}-w_{0}C-w_{t}+w\right) \end{array}$$

where *S* is the crystallized salt mass (g), *V* is the specimen volume (cm^3^), *C* is the brine concentration (%), *w*_0_ is the initial added brine content (%), *w_t_* is the water content at air-drying time *t* (%), and *w* is the natural air-dried water content (%).

By combining Equations (5) and (6), a modified equation incorporating initial added brine content and related specimen conditions was developed with *S* as the independent variable:

(7) $$\begin{array}{l} q={\left(\frac{\rho }{\rho_{s}}\right)}^{m}\cdot \left(ae^{bs}+c\right)\\ ={\left(\frac{\rho }{\rho_{s}}\right)}^{m}\cdot \left(ae^{\frac{b\rho VC}{1-C}\left(w_{0}-w_{0}C-w_{t}+\omega \right)}+c\right)\\ ={\left(\frac{\rho }{\rho_{s}}\right)}^{m}\cdot \left(ae^{bA_{0}\left(w_{0}-w_{0}C-w_{t}+\omega \right)}+c\right) \end{array}$$

where $A_{0}=\frac{\rho VC}{1-C}$, and *m*, *a*, *b*, and *c* are empirical parameters.

**Figure 12:**
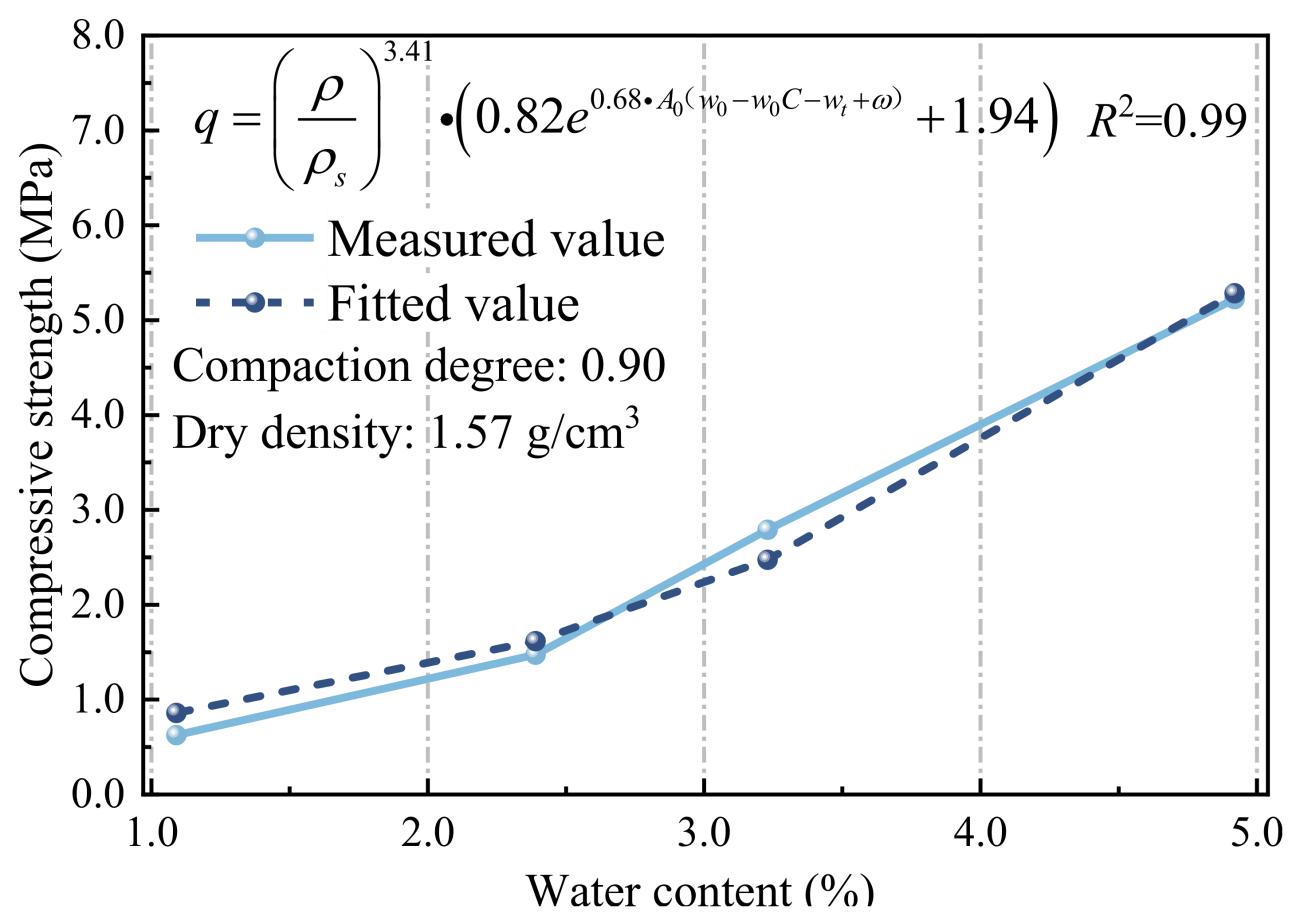
Comparison of measured and fitted UCS values.

Data fitting was performed using the test results of the Group 1 specimens with a degree of compaction of 0.90. The comparison between measured and fitted values is shown in Figure 12, and the fitted strength equation is expressed as follows:

(8) $$q={\left(\frac{\rho }{\rho_{s}}\right)}^{3.41}\cdot \left(0.82e^{0.68A_{0}\left(w_{0}-w_{0}C-w_{t}+\omega \right)}+1.94\right)$$

**Figure 13:**
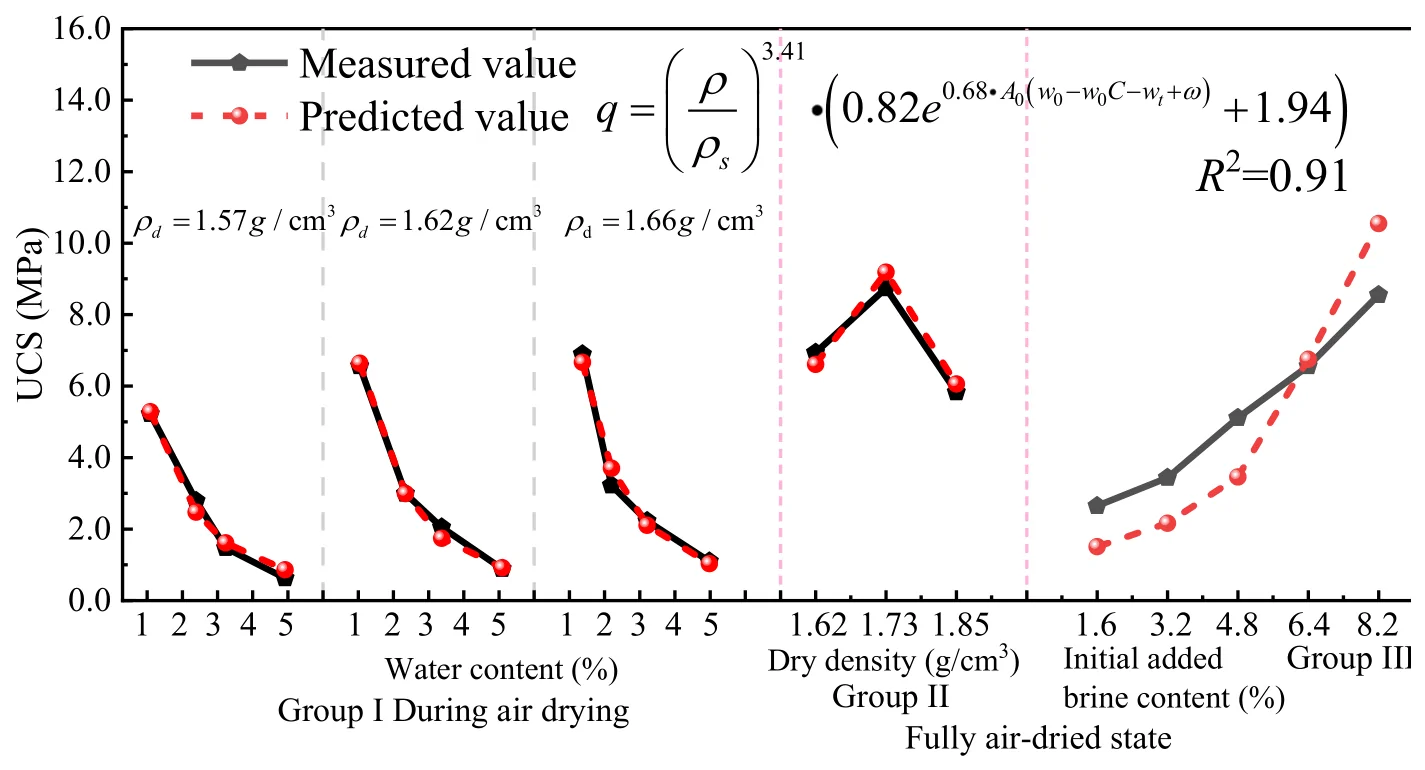
Comparison of measured and predicted UCS values.

The fitting results show that the proposed equation provides a good fit, with *R*^2^ = 0.99. Therefore, it can be used for predicting the UCS of rock salt specimens. The remaining experimental data, including data obtained at different water contents, initial added brine contents, and dry densities, were then used to validate the prediction capability and applicability of the proposed equation. The comparison between measured and predicted UCS values is shown in Figure 13.

The specimens used for model validation were divided into Groups I, II, and III. Group I included specimens with different water contents and dry densities during air drying, as described in Section 3.2. Group II included specimens with different dry densities in the constant-mass air-dried state, as described in Section 3.5. Group III included specimens with different initial added brine contents in the constant-mass air-dried state, as described in Section 3.3.

The results show that, for rock salt specimens prepared under different water contents, dry densities, and initial added brine contents, the values predicted by the proposed equation are in good agreement with the measured values, with *R*^2^ = 0.91. This indicates that the proposed equation is not only suitable for fitting data under different conditions but also has predictive capability across different conditions. Therefore, it is applicable to the prediction of the UCS of rock salt specimens under different influencing factors and parameter levels.

Several points should be noted. First, in Group III, deviations are observed between the measured and predicted values for specimens with different initial added brine contents. For example, the predicted value for the specimen with an initial added brine content of 8.1% is higher than the measured value. This may be related to surface salt efflorescence under a high crystallized salt mass, where not all precipitated salt crystals contribute to effective cementation, resulting in an overestimated prediction. Second, when the initial added brine content is low, the predicted value is slightly lower than the measured value. This may be because, under a low crystallized salt mass, salt crystals are more likely to be locally agglomerated, leading to an uneven spatial distribution of cementation and thus an underestimated prediction. Third, the empirical equation has limited applicability to specimens prepared with unsaturated brine. This is because unsaturated brine can dissolve rock salt particles and dynamically change the pore solution concentration, making the effective crystallized salt mass after air drying difficult to quantify accurately and thereby reducing prediction accuracy. Therefore, the empirical equation established in this study is mainly applicable to predicting the UCS of rock salt specimens mixed with saturated brine.

Cheng et al. [3] previously developed prediction models for the CBR and resilient modulus of rock-salt fillers by considering brine content, the compaction degree, and immersion time. Their work provides an important basis for evaluating the bearing performance and immersion-related behavior of rock-salt subgrade materials. Moreover, the proposed equation is intended to describe UCS evolution during air drying after brine mixing and compaction, rather than CBR or resilient modulus variation under immersion conditions. In this sense, the proposed UCS equation complements the previous CBR/resilient-modulus models by Cheng et al. [3] and provides additional information on drying-induced strength development of compacted rock-salt fills.

### 4.3. Engineering Implications and Applicability to Railway Subgrades

The test results provide preliminary material-level implications for using compacted rock-salt fills in railway subgrades. Under the optimum brine content condition, the UCS of the as-compacted specimen exceeded 0.55 MPa, which is higher than the 7 d UCS requirement for improved soil in the bottom layer of railway subgrades for 160 km/h passenger–freight railways under freeze–thaw cycles, as specified in the *Code for Design of Railway Subgrades* [32]. After drying to constant mass, with a residual water content of approximately 1.1%, the UCS increased to about 6.5 MPa. These results indicate that compacted rock-salt fills can develop a considerable strength reserve under controlled dry conditions, provided that sufficient post-compaction drying is achieved and a low water-content state is maintained.

The effects of brine concentration and the compaction degree should be interpreted cautiously. Lower brine concentration reduced the UCS of dried specimens, suggesting that saturated brine may be more favorable than diluted brine or pure water for preserving salt-related interparticle bonding. However, because statistical significance was not separately demonstrated, this result should be regarded as an observed trend rather than a definitive design rule. Similarly, the apparent optimum compaction degree observed under constant-mass air-dried conditions reflects the combined effects of compactness, residual brine retention, and drying completeness. Therefore, compaction quality control should consider not only dry density, but also drying time, residual water content, and possible recrystallization-induced bonding.

The proposed UCS equation is mainly applicable to rock-salt fills prepared with similar materials, saturated-brine mixing, and controlled air-drying conditions. Direct extrapolation to long-term railway subgrade performance should be avoided, because field subgrades may experience seasonal temperature variation, fluctuating humidity, rainfall infiltration, groundwater intrusion, wetting–drying cycles, salt migration, creep, and repeated traffic loading. These factors may alter dissolution–precipitation behavior and long-term bonding stability. Therefore, the model should be used as a preliminary material-level tool for strength evaluation and construction control, while its extension to long-term railway subgrade design requires durability testing and field validation.

## 5. Conclusions

This study investigated the UCS behavior of compacted rock-salt fills under varying water content, initial brine content, dry density, compaction degrees, and brine concentration. The main conclusions are as follows:

(1) Under the laboratory air-drying conditions of 25–28 °C and 20–30% relative humidity, specimens prepared at the optimum brine content and a compaction degree of 0.93 reached a nearly constant-mass state after approximately 10 days, with a residual water content of about 1.1%. The UCS increased from 0.89 MPa in the as-compacted state to approximately 6.5 MPa after drying, indicating substantial drying-induced strength gain.
(2) UCS increased nonlinearly as water content decreased. The strength increase was limited at relatively high water contents, but accelerated markedly when the water content decreased to approximately 1.0–2.3%. This behavior is consistent with a transition from capillary liquid bridges to stronger salt-related interparticle bonding during brine evaporation and possible recrystallization.
(3) The relative influence of the investigated factors followed the order of water content > initial added brine content > dry density or compaction degree > brine concentration, with corresponding range values of 5.67, 5.09, 3.52, and 1.10 MPa. Water content was therefore the dominant factor controlling UCS within the tested range, whereas brine concentration showed the weakest influence.
(4) Initial brine content had opposite effects before and after drying. In the as-compacted state, increasing the initial brine content from 1.6% to 8.1% reduced UCS by about 63%, mainly because of lubrication and weakened particle friction. After drying to constant mass, UCS increased by about 223% over the same brine-content range, suggesting that higher brine addition supplied more crystallizable salt for subsequent interparticle bonding.
(5) Under constant-mass air-dried conditions, UCS first increased and then decreased with an increasing compaction degree, reaching a peak of approximately 8.7 MPa at a compaction degree of 0.99 and a dry density of about 1.72–1.73 g/cm^3^. The lower strength at higher compaction was associated with residual brine retention and incomplete drying; after oven drying, UCS increased with the compaction degree, indicating that both compactness and drying completeness affect the final strength.
(6) Lower brine concentration reduced the UCS of dried specimens, with decreases of approximately 10.9% for half-saturated brine and 16.6% for pure water compared with saturated brine at 24.95 wt%. The proposed empirical equation predicted the measured UCS values with *R*^2^ = 0.91. However, the conclusions and prediction model are mainly applicable to the tested material and controlled dry laboratory conditions. Future studies should provide direct microstructural verification and evaluate long-term performance under rainfall infiltration, wetting–drying cycles, salt migration, creep, repeated traffic loading, and field-scale environmental variations.

## Figures and Tables

**Table 1:** Semi-quantitative XRD mineral composition of rock salt and evaporated brine crystals (%).

Category	Halite	K-Bearing Salts	Calcite	Plagioclase	Quartz	Hemihydrate Gypsum	Chlorite	Gypsum	Illite
Rock salt	79.3	4.9	2.0	1.5	4.3	1.5	2.3	1.6	2.6
Evaporated crystals from saturated brine	98.4	1.6	—	—	—	—	—	—	—

Note: “—” indicates not detected. The values are semi-quantitative XRD results and represent approximate phase proportions. Minor phases with weak or overlapping diffraction peaks may not be fully identified.

**Table 2:** UCS test matrix.

No.	Initial Brine Content (%)	Compaction Degree	Brine Concentration (wt%)	Air-Drying Duration	Testing State
1	6.4	0.90, 0.93, 0.95	24.95	0, 1, 3, and 7 d; const. mass	Air-dried at different durations
2	1.6, 3.2, 4.8, 6.4, 8.1	0.93	24.95	0 d; const. mass	As-compacted and const.-mass air-dried
3	6.4	0.93, 0.99, 1.06	24.95	Const. mass	Const.-mass air-dried
4	6.4	0.93	0, 12.48, 24.95	Const. mass	Const.-mass air-dried

Note: “Const. mass” refers to the air-dried state in which the specimen mass change was ≤0.1% within 24 h.

**Table 3:** Measured and mass-balance-calculated water contents under different air-drying durations.

Compaction Degree	Air-Drying Time (d)	Measured Water Content (%)	Mass-Balance-Calculated Water Content (%)	Difference (Measured—Theoretical, %)	Relative Difference (%)
0.90	0	4.92	4.66	0.26	5.28
0.93		5.09	4.76	0.33	6.48
0.95		4.98	4.84	0.14	2.81
0.90	1	3.23	2.93	0.30	9.29
0.93		3.37	3.16	0.21	6.23
0.95		3.21	3.27	−0.06	−1.87
0.90	3	2.39	1.84	0.50	23.01
0.93		2.34	1.91	0.43	18.38
0.95		2.20	2.00	0.20	9.09
0.90	7	1.09	1.29	−0.20	−18.35
0.93		1.04	1.25	−0.21	−20.19
0.95		1.37	1.19	0.18	13.14
0.90	Const. mass (10)	1.20	1.34	−0.10	−11.67
0.93		1.11	1.26	−0.15	−13.51
0.95		1.29	1.29	−0.04	0.00

**Table 4:** Fitted parameters and 95% confidence intervals of the exponential UCS–water content relationships.

Compaction Degree	Parameter a	95% CI of a	Parameter b	95% CI of b	Parameter c	95% CI of c	*R* ^2^
0.90	9.43	(−0.41, 19.27)	0.49	(−1.23, 2.22)	−0.27	(−9.29, 8.76)	0.98
0.93	11.76	(−2.52, 26.06)	0.64	(−0.95, 2.24)	0.52	(−5.11, 6.15)	0.97
0.95	26.03	(−103.42, 155.49)	1.11	(−2.82, 5.04)	1.16	(−4.91, 7.22)	0.99

## Data Availability

The original contributions presented in this study are included in the article. Further inquiries can be directed to the corresponding author.
